# Supplementary material for: Rare but elevated incidence of hematological malignancy after clozapine use in schizophrenia: A population cohort study
Source: PLoS Med. 2024 Dec 5;21(12):e1004457. doi: 10.1371/journal.pmed.1004457 (PMC11620352; doi:10.1371/journal.pmed.1004457)
Supplement: S1 Table — (DOCX) [file pmed.1004457.s002.docx]

| S1 Table. ICD-9-CM diagnostic codes and generic drug names used to define covariates | |
| --- | --- |
| Disease name/ Drug category | ICD-9-CM codes/ drug names |
| Hematological malignancy |  |
| Lymphosarcoma and reticulosarcoma | 200 |
| Hodgkin's disease | 201 |
| Other malignant neoplasms of lymphoid and histiocytic tissue | 202 |
| Multiple myeloma and immunoproliferative neoplasms | 203 |
| Lymphoid leukaemia | 204 |
| Myeloid leukaemia | 205 |
| Monocytic leukaemia | 206 |
| Other specified leukaemia | 207 |
| Leukaemia of unspecified cell type | 208 |
| Polycythemia vera | 238.4 |
| Neoplasm of uncertain behavior of histiocytic and mast cells | 238.5 |
| Neoplasm of uncertain behavior of plasma cells | 238.6 |
| Neoplasm of uncertain behavior of other lymphatic and hematopoietic tissue | 238.7 |
| Mental illnesses |  |
| Depression | 296.2, 296.3, 300.4, 625.4, 293.83, 311, 648.4, 300.0 |
| Bipolar disorder | 296 |
| Dementia | 290, 294.1, 294.2, 294.8, 331.0, 331.1, 331.82 |
| Immunological disease | 42, 43, 44, 266.2, 273.0, 273.1, 273.2, 277.2, 277.6, 279, 288.1, 289.89 |
| Autoimmune Diseases: |  |
| Vitiligo | 709.01 |
| Addison disease | 255.4 |
| Alopecia areata | 704.01 |
| Autoimmune/Hashimoto’s thyroiditis | 245.2 |
| Graves’ disease | 242 |
| Morphoea | 701 |
| Multiple sclerosis | 340 |
| Myasthenia gravis | 358 |
| Pernicious anaemia | 281 |
| Primary biliary cirrhosis | 571.6 |
| Takayasu arteritis | 446.7 |
| Type 1 diabetes mellitus | 250.X1, 250.X3 |
| Associated Conditions: |  |
| Episcleritis/scleritis | 379.0X |
| Erythema nodosum | 695.2 |
| Haemolytic anaemia | 283.XX |
| Immune thrombocytopenia purpura | 287.31 |
| Leukocytoclastic vasculitis | 446.29 |
| Myositis | 729.1 |
| Pulmonary fibrosis/interstitial lung disease | 515. X, 516.31 |
| Raynaud’s syndrome | 443 |
| Sjögren’s syndrome/sicca syndrome | 710.2 |
| Thrombocytopenia purpura | 287.3 |
| Vasculitis | 447.6 |
| Juvenile idiopathic arthritis (JIA) | 714.30, 714.31, 714.32, 714.33, 714.2 |
| Acquired immune deficiencies | 42, 43, 44 |
| Asthma | 493 |
| Previous antipsychotic use |  |
| Phenothiazines | Chlorpromazine, Perphenazine, Fluphenazine, Trifluoperazine, Pericyazine, Thioridazine |
| Thioxanthene derivatives | Flupenthixol, Zuclopenthixol, Thiothixene |
| Diphenylbutylpiperidine derivatives | Pimozide |
| Butyrophenone derivatives | Haloperidol, Droperidol |
| Benzamides | Sulpiride, Amisulpride |
| Indole derivatives | Lurasidone, Ziprasidone, Molindone, Sertindole |
| Diazepines, oxazepines, thiazepines and oxepines | Quetiapine, Asenapine |
| Other antipsychotics | Aripiprazole, Brexpiprazole, Risperidone, Paliperidone |
