# Supplementary material for: Rare but elevated incidence of hematological malignancy after clozapine use in schizophrenia: A population cohort study
Source: PLoS Med. 2024 Dec 5;21(12):e1004457. doi: 10.1371/journal.pmed.1004457 (PMC11620352; doi:10.1371/journal.pmed.1004457)
Supplement: S2 Table — (DOCX) [file pmed.1004457.s003.docx]

| S2 Table. Sensitivity analysis: incidence rate ratios (IRR) of hematological malignancy (HM) with 95% confidence intervals (CI) | | | | | | | | | |
| --- | --- | --- | --- | --- | --- | --- | --- | --- | --- |
|  | Cohort size | Average follow-up duration (days) | Number of HM cases | Crude incidence rate of HM per 100,000 person-year | Weighted incidence rate of HM per 100,000 person-year | Crude IRR  (HR for Cox regression) [95% CI] | Weighted IRR  (HR for Cox regression) [95% CI] | Weighted absolute rate difference per 100,000 person-years [95% CI] |  |
| Clozapine or olanzapine users of 180+ days | 6,091 | 2781.40 | 21 | 45.27 | 59.46 |  |  | 34.79 [6.94, 62.65] |  |
| Olanzapine | 5,529 | 2618.33 | 16 | 40.37 | 38.85 | Ref. | Ref. |  |  |
| Clozapine | 562 | 4385.72 | 5 | 74.09 | 73.65 | 1.84 [0.6, 4.69] | 2.29 [1.29, 4.07] |  |  |
| Right-censoring at 3 years of treatment discontinuation | 9,965 | 2709.44 | 39 | 52.76 | 79.99 |  |  | 56.53 [31.76, 81.29] |  |
| Olanzapine | 9,131 | 2546.58 | 30 | 47.12 | 45.79 | Ref. | Ref. |  |  |
| Clozapine | 834 | 4492.47 | 9 | 87.74 | 102.32 | 1.93 [0.86, 3.91] | 2.22 [1.32, 4.19] |  |  |
| Multivariable Poisson regression | 9,965 | 2821.47 | 39 | 50.66 | 78.01 |  |  | - |  |
| Olanzapine | 9,131 | 2664.43 | 30 | 45.04 | 43.75 | Ref. | Ref. |  |  |
| Clozapine | 834 | 4540.90 | 9 | 86.80 | 101.15 | 1.93 [0.86, 3.90] | 2.53 [1.04, 5.42] |  |  |
| Weighted Cox regression | 9,965 | 2821.47 | 39 | 50.66 | 78.01 |  |  | 57.40 [33.24, 81.55] |  |
| Olanzapine | 9,131 | 2664.43 | 30 | 45.04 | 43.75 | Ref. | Ref. |  |  |
| Clozapine | 834 | 4540.90 | 9 | 86.80 | 101.15 | 1.95 [0.91, 4.19] | 2.28 [0.80, 6.48] |  |  |
| Using asthma as negative control outcome | 9,814 | 2817.21 | 74 | 97.76 | 106.17 |  |  | 8.73 [0.00, 37.93] |  |
| Olanzapine | 8,989 | 2660.68 | 64 | 97.74 | 100.30 | Ref. | Ref. |  |  |
| Clozapine | 825 | 4522.73 | 10 | 97.89 | 110.20 | 1.01 [0.52, 1.97] | 0.90 [0.66, 1.21] |  |  |
| Using other cancers as negative control outcome | 9,982 | 2747.85 | 597 | 794.98 | 644.99 |  |  | 175.53 [97.68, 253.3] |  |
| Olanzapine | 9,140 | 2595.88 | 497 | 765.1 | 749.89 | Ref. | Ref. |  |  |
| Clozapine | 842 | 4397.43 | 70 | 690.52 | 574.35 | 0.90 [0.70, 1.15] | 0.77 [0.68, 0.86] |  |  |
| Excluding 50% clozapine users with lowest daily dose | 9547 | 2801.47 | 33 | 45.07 | 104.64 |  |  | 96.68 [70.24,123.12] |  |
| Olanzapine | 9130 | 2664.71 | 30 | 45.04 | 44.07 | Ref. | Ref. |  |  |
| Clozapine | 417 | 4796.16 | 3 | 54.79 | 140.75 | 1.17 [0.28,3.28] | 3.16 [2.17,4.78] |  |  |
| Excluding 25% clozapine users with lowest daily dose | 9756 | 2766.23 | 36 | 48.72 | 88.76 |  |  | 73.54 [73.54,98.61] |  |
| Olanzapine | 9130 | 2664.71 | 30 | 45.04 | 43.75 | Ref. | Ref. |  |  |
| Clozapine | 626 | 4989.08 | 6 | 70.17 | 117.28 | 1.62 [0.61,3.63] | 2.93 [2.00,4.42] |  |  |
| Patients having used ≥ 3 different antipsychotics | 3861 | 3064.78 | 19 | 58.65 | 70.68 |  |  | 40.42 [3.66, 77.18] |  |
| Olanzapine | 3365 | 2838.40 | 13 | 49.71 | 46.85 | Ref. | Ref. |  |  |
| Clozapine | 496 | 4600.59 | 6 | 96.04 | 87.28 | 1.93 [0.68, 4.89] | 1.88 [1.05, 3.57] |  |  |
| Truncating extreme weights out of 1st – 99th percentile | 9965 | 2821.47 | 39 | 50.66 | 75.59 |  |  | 56.64 [31.45, 81.84] |  |
| Olanzapine | 9131 | 2664.43 | 30 | 45.04 | 43.75 | Ref. | Ref. |  |  |
| Clozapine | 834 | 4540.9 | 9 | 86.80 | 100.4 | 1.93 [0.86, 3.90] | 2.31 [1.34, 8.94] |  |  |
