# Supplementary material for: Rare but elevated incidence of hematological malignancy after clozapine use in schizophrenia: A population cohort study
Source: PLoS Med. 2024 Dec 5;21(12):e1004457. doi: 10.1371/journal.pmed.1004457 (PMC11620352; doi:10.1371/journal.pmed.1004457)
Supplement: Attachment — Submitted filename: Response Letter_final.docx [file pmed.1004457.s004.docx]

September 25^th^, 2024

Dr. Alexandra Tosun

Associate Editor, *PLOS Medicine*

Dear Dr. Tosun,

Thank you very much for considering our manuscript favorably and giving us the opportunity to strengthen it further. We are very grateful for the insightful reviews relayed from the reviewers and the editorial team’s comments. Please find appended below our detailed response to each of the specific comments with proposed changes and additional analyses. The revised relevant text has been quoted as appropriate:

-----------------------------------------------------------

**Editorial Team**

**E1.	“The manuscript generated a lot of discussion among the editorial team. There were some concerns about the manuscript and whether it is of sufficient general interest for MED, given the rarity of the condition and treatment. We encourage you to expand on why the study will be of interest to a general health readership and have public health relevance in the introduction and discussion.”**

**Author Response:**

Thank you for this important suggestion and opportunity to clarify. In line with the reviewers’ comments, there is indeed a general consensus in psychiatry that clozapine is generally safe and highly efficacious with a unique mechanism, but substantially underused largely due to the need for blood monitoring. In fact, it is commonly recognized as the strongest antipsychotic drug currently available. Thus, it has long been encouraged that clozapine be used in much earlier phases of schizophrenia for early intervention and for more patients. With improved understanding of its actions and safety profile in recent years, clozapine indeed has the potential to become one of the most widely used psychotropic drugs in the foreseeable future. Some existing studies even suggest a potentially good effectiveness in bipolar disorder and other non-schizophrenic disorders. Our study is in alignment with this advocacy and trend by demonstrating that although the elevated risk of hematological malignancy shown in two previous nationwide case-control studies is likely true, the absolute rate difference is too small to be overly concerned, given existing risk mitigation measures. Therefore, the rarity of the outcome event, i.e., hematological malignancy, in this study does not make the study less relevant. Rather, it helps build a stronger case for a much wider use of clozapine for early intervention as a crucial instrument to improving population mental health. In the revised manuscript, we have now elaborated on the potentials of clozapine to become one of the most important psychotropic drugs in the near future and how our study contributes to its evidence base, as quoted below.

“*Clozapine is widely regarded as a safe and highly efficacious psychotropic drug that is largely underused worldwide.*”

(Lines 39-40, Page 2)

“*Clozapine is a second-generation antipsychotic agent widely regarded as a safe and highly efficacious psychotropic drug, with the lowest all-cause mortality rate observed compared with other antipsychotics (1, 2). It is largely considered underused worldwide, possibly due to blood monitoring requirements (3).*”

(Lines 89-92, Page 4)

“*In the existing literature, there is a consensus that clozapine is safe, highly efficacious, but largely underused worldwide (1), possibly due to the need for blood monitoring (3). It is widely encouraged to use it in an earlier phase of psychosis and possibly for more patients (32). Studies have suggested its potential efficacy for patients with bipolar disorder and other mental disorders (33). With an enhanced understanding of its pharmacological actions and safety profile in recent years, clozapine indeed has the potential to become one of the most important psychotropic drugs in the world and act as an important instrument for safeguarding population mental health in the foreseeable future. In line with this development, this study is of great importance because it determines the clinical significance of the potential risk of HM by providing reassuring data on a small, barely concerning elevated risk. Given the existing strict requirements for blood monitoring which helps early identify HM development and the rarity of HM, the risk-benefit balance for clozapine pharmacotherapies remains largely unchanged. Subject to further studies to substantiate our findings, special warnings or more restrictive indication for clozapine may not be necessary.*”

(Lines 310-323, Page 9)

-----------------------------------------------------------

**Reviewer 1:**

**1.1.	“This manuscript deals with a very important clinical issue, the risk of severe adverse effect attributable to clozapine treatment. Although two previous studies have been published earlier on this issue, reporting similar results on the risk increase of hematological cancer, this study is a valuable contribution. It confirms the previous findings, but has a slightly different methodology, which made it possible to estimate the antipsychotic exposure lead prior to two treatments (clozapine vs. olanzapine) in this study. I have a few comments:”**

**Author Response:**

Thank you very much for recognizing the value of our study.

**1.2.	“The authors conclude in the Abstract that "Absolute rate difference in HM incidence was very small although…" Since patients are typically treated with clozapine for 10-20 years or even longer, it can be estimated that absolute rate difference of 57.4 per 100,000 persons years would be (57.4/100,000) x 15 = 0.9% during a follow-up of 15 years. This is the same cumulative incidence which has been observed for agranulocytosis. Since mortality related to hematological cancer is higher than that related to agranulocytosis (which is rather low because of early detection and growth factor treatments), it can be estimated that excess hematological cancers probably contribute more to mortality than agranulocytosis among patients treated with clozapine. So, the wording "very rare" might be reconsidered.”**

**Author Response:**

Thank you for this comment. It is true that if clozapine is used over a long duration, which is indeed typical, the cumulative risk of hematological malignancy will be much higher than the value of the absolute rate presented because the latter only represents the average risk per year, not cumulative. We have now inserted in the revised text a brief reminder on the interpretation of this absolute rate in users with long duration of use. Although we checked and found that we did not use ‘very rare’ as a label in the manuscript, we changed our expression in the abstract from “very small” to just “small” to avoid understating the absolute risk by presenting the rate.

“*Absolute rate difference in HM incidence associated with clozapine is small despite a twofold elevated rate. Given the rarity of HM and existing blood monitoring requirements, more restrictive indication for clozapine or special warnings may not be necessary.*”

(Lines 59-61, Page 2)

“*Although the absolute rate difference is small, a very long duration of clozapine use, which is not uncommon, may constitute a nonnegligible cumulative risk of HM.*”

(Lines 301-303, Page 8)

**1.3.	“The conclusions discuss clinical implications, stating that psychiatrists may be more inclined to try other antipsychotics before initiating clozapine. I think that the psychiatrists should do exactly the opposite: use clozapine more and earlier. The authors should acknowledge that since clozapine is associated with substantially lower risk of death (due to antisuicidal etc. effects) than other antipsychotics (i.e., it is the safest antipsychotic treatment in terms of mortality, ref. Correll et al. Mortality in people with schizophrenia: a systematic review and meta-analysis of relative risk and aggravating or attenuating factors. World Psychiatry. 2022 Jun;21(2):248-271. doi: 10.1002/wps.20994), it should be used earlier than what is currently done. I think that early detection of the possible hematological cancers is the main implication: patients and families should be informed about warning signs, and clinicians should be vigilant (e.g., about one third of the patients developing lymphoma have anemia before diagnosis, and this could be observed in mandatory blood monitoring of patients with clozapine).”**

**Author Response:**

Thank you for this helpful comment. We do agree that clozapine is overall a safe, highly efficacious, and oftentimes superior, option over other antipsychotics for patients with schizophrenia. We also agree it should be used earlier than actual current practices to achieve better treatment outcomes. We realize the original paragraph on clinical implications was slightly more focused on how the findings of an elevated risk of hematological malignancy following clozapine use might lead to clinicians’ and patients’ hesitancy towards clozapine prescription instead of how the implications should be correctly interpreted jointly with existing safety and effectiveness evidence. We have now modified the paragraph to highlight the safety and effectiveness of clozapine and the trend of earlier and wider use of clozapine and how the small rate difference of HM observed should not be overly concerning.

“*In the existing literature, there is a consensus that clozapine is safe, highly efficacious, but largely underused worldwide (1), possibly due to the need for blood monitoring (3). It is widely encouraged to use it in an earlier phase of psychosis and possibly for more patients (32). Studies have suggested its potential efficacy for patients with bipolar disorder and other mental disorders (33). With an enhanced understanding of its pharmacological actions and safety profile in recent years, clozapine indeed has the potential to become one of the most important psychotropic drugs in the world and act as an important instrument for safeguarding population mental health in the foreseeable future. In line with this development, this study is of great importance because it determines the clinical significance of the potential risk of HM by providing reassuring data on a small, barely concerning elevated risk. Given the existing strict requirements for blood monitoring which helps early identify HM development and the rarity of HM, the risk-benefit balance for clozapine pharmacotherapies remains largely unchanged. Subject to further studies to substantiate our findings, special warnings or more restrictive indication for clozapine may not be necessary.*”

(Lines 310-323, Page 9)

“*To conclude, we conducted a territory-wide retrospective cohort study in Hong Kong and identified a rare but evidently elevated risk of HM among clozapine users with schizophrenia compared with olanzapine users. With a small rate difference and existing blood monitoring requirements, no special warnings or tightening of indication are strictly necessary or recommended. Clinicians should be aware of this consistently observed association but noting its rarity while weighing it against the benefits of clozapine pharmacotherapies.*”

(Lines 359-364, Page 10)

-----------------------------------------------------------

**Reviewer 2:**

**2.1.	“Thanks for the opportunity to read your manuscript. My role is statistical reviewer, so I have focused on the design, data, and analysis that are presented. I have put general comments first, followed by questions relevant to a specific section of the manuscript (with a page/line reference). The manuscript examines if there is evidence that exposure to clozapine puts people with schizophrenia at higher risk of blood cancers. Data is the from the linked healthcare data from Hong Kong, which links diagnoses from inpatient and outpatient records, with medication dispensing records. Participants were identified from a recorded diagnosis of schizophrenia, and use of either clozapine or olanzapine for at least 90 days (first day of this period is the inception date) between 2001 and Aug 2022, with a gap of less than 7 days in coverage ignored for defining the 90-day period. Outcomes were all haematological malignancies recorded in the in and outpatient data. A lookback period of up to 1999 for previous exposure to clozapine/olanzapine, other antipsychotics, and previous HM diagnosis was used. Patients were followed until diagnosis, death, end of data, or up to 5 years from inception. The main analysis used inverse-probability weights, with probability derived from a logistic regression model that included age, sex, previous use of antipsychotics, mental health history, and autoimmune disease. The main analysis used Poisson regression to estimate IRR, with an offset term for differing follow-up periods. Sub-group analyses by age and sex were included, and several sensitivity analyses that considered the effect of a longer period of exposure for inclusion, shorter follow-up, a multivariable model instead of IPW, cox regression, and negative control (asthma and other cancers as outcome). The IPW, the choice of patients exposed to olanzapine as the comparator, and use of a negative control are all robust approaches for this research question. A relatively large cohort (9965 with 834 clozapine users) was selected, and good balance achieved with IP weights. There is a small but consistent difference in absolute rate of HM between olanzapine and clozapine users. The sensitivity analyses were consistent with the main results, with a small difference in absolute rate of the negative control outcomes for other cancers.”**

**Author Response:**

Thank you very much for your encouraging comments on the robustness of the approaches adopted.

**2.2.	“P4, L140. Were there any extreme weights? If so, how were these dealt with? Was there evidence of common support? This could be demonstrated with a figure showing the distribution of the propensity score by exposure group.”**

**Author Response:**

Thank you for this suggestion. We have constructed a figure (not for publication) showing the distribution of the assigned weights with notable common support and no obvious extreme values, as shown below. In addition, we repeated the main analysis adopting the truncation approach to use only weights within the range of 1^st^ – 99^th^ percentile and did not find a markedly different incidence rate ratio (S2 Table).

“… *vi) top and lowest 1% of the weights generated with propensity scores were truncated in a repeated main analysis despite the absence of obvious outliers…*”

(Lines 194-196, Page 6)

**2.3.	“P4, L145. Was there any missing covariate data? If so, what approach was used?”**

**Author Response:**

Thank you for this comment. As shown in Figure 1, there were no missing data on demographic information, i.e., age and sex, for which we would have excluded patients. For covariates such as chronic conditions and medications, we adopted a similar approach as other observational research using electronic health records and assumed the absence of a disease or medication if no relevant record was identified for the patients. We have now stated this approach in the Methods section for better clarity.

“*Similar with other observational electronic health record studies, we assumed the absence of a disease or medication if no relevant record was identified for the patients in the database. Those without basic demographic information, i.e., age and sex, would be excluded.*”

(Lines 201-203, Page 6)

**2.4.	“P5, L158. What approach was used for the subgroup analyses? Stratification by subgroup variables, or an interaction term?”**

**Author Response:**

Thank you for this comment. We stratified the sample by subgroup variables without testing for the interaction term because we did not hypothesize a different association across subgroups.

**2.5.	“P5, L158. What was the rationale of the sensitivity analyses using a multivariable model, and a Cox model?”**

**Author Response:**

Thank you for the opportunity to clarify the rationale of the sensitivity analyses. We conducted the analysis using Cox model or multivariable Poisson to enhance the comparability with other studies which may be using slightly different statistical methods than our main analysis. This sensitivity analysis also demonstrates the robustness of the results across different statistical methods. We have now explained the rationale briefly in the revised Methods section.

“*… iii) to enhance the comparability with other studies which may be using slightly different statistical methods than our main analysis, a multivariable Poisson regression to employed to replicate the analysis; iv) likewise, a Cox regression with the IPTW approach was used to estimate the hazard ratio…*”

(Lines 188-191, Page 6)

**2.6.	“P5, L164. What software was used?”**

**Author Response:**

We used the R statistical programming environment (version 4.1.2) for all analyses. We have now stated it clearly in the revised text.

“*Similar with other observational electronic health record studies, we assumed the absence of a disease or medication if no relevant record was identified for the patients in the database. Those without basic demographic information, i.e., age and sex, would be excluded. YH and LG were independent analysts of this study to minimize errors in coding and programming. The R statistical programming environment (version 4.1.2) was used to conduct all the described analyses, with the ‘survminer’ package used to build cumulative incidence plot. Statistical tests were two-tailed and a P value of 0.05 or smaller was indicative of statistical significance.*”

(Lines 201-207, Page 6)

**2.7.	“P5, L189. What approach was used to generate the IP weighted cumulative incidence curves?”**

**Author Response:**

The function “ggsurvplot” of R package “survminer” was used to generate the curves.

“*Similar with other observational electronic health record studies, we assumed the absence of a disease or medication if no relevant record was identified for the patients in the database. Those without basic demographic information, i.e., age and sex, would be excluded. YH and LG were independent analysts of this study to minimize errors in coding and programming. The R statistical programming environment (version 4.1.2) was used to conduct all the described analyses, with the ‘survminer’ package used to build cumulative incidence plot. Statistical tests were two-tailed and a P value of 0.05 or smaller was indicative of statistical significance.*”

(Lines 201-207, Page 6)

**2.8.	“P6, L206. How should the IRR of for the negative control for all cancers be interpreted? Is this likely to be due to residual confounding?”**

**Author Response:**

Thank you for this comment. The purpose of including negative control outcomes as a sensitivity analysis is to detect potential biases or confounders in research. The formal definition of a negative control outcome is one that shares the same potential sources of bias with the primary outcome but cannot plausibly be related to the treatment of interest (reference). The idea is that if an association is observed between the exposure and the negative control outcome, the observed association of clozapine with hematological malignancy is likely due to bias or confounding factors. An incidence rate ratio of all other cancers significantly smaller than one as observed in this sensitivity analysis may indeed suggest other factors at play. For instance, clozapine has been suggested to be potentially protective of certain types of cancers in cell line studies, which may require further studies to substantiate. We have now briefly discussed this finding in the revised text.

“*Also, the potential reasons for the IRR estimated for all other cancers being significantly lower than one requires further research to investigate, which may include potential protective effect of clozapine on certain types of cancer suggested by previous cell line studies (31).*”

(Lines 306-308, Page 9)

-----------------------------------------------------------

**Reviewer 3:**

**3.1.	“This is an important study adding knowledge to the queston of higher risk of haemtological malignancies (HM) in clozapine users compared to other antipsychotics (in this study olanzapine). Especially the calculation of the absolute rate difference of HM is very useful for clinical decision making. The study uses a cohort design which strenghtens the findings of earlier case-control studies. I have some suggestions for additoinal analyses and some comments on the authors'conclusion.”**

**Author Response:**

Thank you very much for your positive comment on the importance of this study.

**3.2.	“1) the authors state: more clozapine users were prescribed more than three kinds of other antipsychotics in the past year (59.5% versus 36.9%). this is not astonishing since cozapine often is prescribed after numerous trials of different antipsychotics and polypharmacy. I would suggest to make an additional analysis of clozapine and olanzapine users who used three or four or five different antipsychotics prior to the use of clozapine/olanzapine. This could strengthen the point that both groups were therapy-resistant, but that the olanzapine group did not receive clozapine despite a probable indication for it.”**

**Author Response:**

Thank you for this suggestion. In the main analysis, the patients had a prior record of using two or more antipsychotics. We have now conducted an additional sensitivity analysis to include only those who used three or more different antipsychotic drugs and obtained similar results with the incidence rate ratio estimated at 1.88 (95% CI 1.05. 3.57).

“… *viii) we further confined the sample to those who used three or more antipsychotics prior to clozapine or olanzapine use and repeated the analysis.*”

(Lines XXX, Page XXX)

**3.3.	“2) Tiihonen et al. (Lancet Psychiatry 2022) showed that clozapine use was associated with increased odds of haematological malignancies in a dose-response manner. It would strengthen the author's case of a causal relationshiop between clozpaine and HM if they could show this association too. Also, Tiihonen found that longer but not shorter use than 5 years of clozapine was associated with an increased risk. Do the authors find the same? ”**

**Author Response:**

Thank you for this excellent suggestion. We have now calculated the average prescribed daily dose for all clozapine users over their duration of clozapine use. Subsequently, we conducted two additional sets of sensitivity analyses by excluding the 25% and 50% of clozapine users with the lowest average daily doses from the sample. Our observations indicated a potential dose-response relationship, with incidence rate ratio estimated at 2.93 (95% CI [2.00, 4.42]) and 3.16 (95% CI [2.17,4.78]), respectively. The comparison of prior duration of clozapine use in terms of the risk of HM, e.g., 5+ versus 0-4 years, is typically only conducted in case-control studies with a retrospective ascertainment of drug use but not in cohort studies with more strictly defined timeframes for the ascertainment of drug use. As shown in Figure 2, we do not observe a certain point during the follow-up period at which the incidence of HM shoots up. The cumulative incidence was consistently higher among clozapine users than among olanzapine users.

“… *vii) we excluded one-fourth, and half of the clozapine users who had the lowest prescribed daily dose average across the duration of use in a repeated set of main analysis to observe any potential dose-response relationship…*”

(Lines 196-198, Page 6)

“*With 25% or 50% of clozapine users with the lowest prescribed daily dose average excluded, we estimated larger IRRs which indicate a potential dose-response relationship, i.e., 2.93 (95% CI [2.00, 4.42]) and 3.16 (95% CI [2.17,4.78]). The median average prescribed daily dose of clozapine was 581.57 mg/day and 13.72 mg/day for olanzapine.*”

(Lines 249-253, Page 7)

“*Subgroup analyses by age and sex, as well as sensitivity analyses, showed highly robust results with a potential dose-response relationship, supporting a comparably strong and highly specific association. Our research hypothesis is therefore well supported by the data.*”

(Lines 262-265, Page 8)

**3.3.	“I have some comments for the discussion/conclusion. The authors state: This potential slight recalibration of the risk-benefit balance may partially impact clinical decisions about long-term clozapine use. I strongly advise to put the findings in a more cautious perspective. Prescribers and patients perceive clozapine as a hazardous drug which prevents appropriate life-saving use in many patients (Verdoux et al. Prescriber and institutional barriers and facilitators of clozapine use: A systematic review, Schizophrenia Research 2018). The authors should stress that although clozapine has this higher risk of HM it is still the antipsychotic with the lowest mortality (Taipale, H., Tanskanen, A., Meht¨al¨a, J., et al., 2020. 20-year follow-up study of physical morbidity and mortality in relationship to antipsychotic treatment in a nationwide cohort of 62,250 patients with schizophrenia (FIN20). World Psychiatry 19, 61-68.) Tiihonen et al. (2022) state: "Although the absolute risk for haematological malignancy among patients who had used clozapine was 0·7% during the mean of 12·3 years of follow-up, the absolute risk reduction for clozapine versus other antipsychotic drugs in all-cause mortality in the same cohort was substantially greater (about 10%), in line with a previous meta-analysis." Furthermore, they found an improved prognosis of HM in clozapine users (32∙9% mortality in patients with ongoing clozapine use vs 50∙7% in non-clozapine users in the investigated cohorts). In the light of these finding the author's advices from line 261 up to 268 are not justified (first advice), certainlyl if the higher risk of individuals with significant hematological abnormalities or a family history of HM cannot be quantified. At least should the authors state that in the light of the low overall mortality of clozapine a higher risk in these groups should be weighed against the lower overall mortality and the the hgher quality adjusted life years with clozapine. This remains the realm of shared decision making. The authors state rightly that their "potential considerations need to be supported by additional evidence" adnmitting that their advice is premature. I suggest that the authors conclude that the absolute rate differecne vor HM between clozapine and olanzapine users is very small: (57.40 (95% CI 33.24, 81.55) per 100,000 person-years) and in the light of the very low overall mortality of clozapine in comparison to other antipsychotics is reassuring and should not lead to more restrictive indication of clozapine nor special warnings of patients of this slight additional risk. Prescribers should know this potential complication of clozapine therapy and recognize symptoms and signs. They may choose to control in addition to the granulocytes the leucocytes, differential, erythrocytes en thrombocytes once per year.”**

**Author Response:**

Thank you for this helpful comment. We do agree that clozapine is overall a safe, highly efficacious, and oftentimes superior, option over other antipsychotics for patients with schizophrenia. We realize the original paragraph on clinical implications was slightly more focused on how the findings of an elevated risk of hematological malignancy following clozapine use might potentially lead to clinicians’ and patients’ hesitancy towards clozapine prescription instead of how the implications should be correctly interpreted jointly with existing safety and effectiveness evidence. We have now modified the paragraph to highlight the benefits of earlier and wider use of clozapine, specifically the overall comparative safety and effectiveness in terms of mortality and quality-adjusted life years. In addition, we have cited the previously observed better prognosis of hematological malignancy among clozapine users compared with non-users. Given the small absolute rate difference, we further stated that a more restrictive indication of clozapine or special warnings may not be necessary.

“*In the existing literature, there is a consensus that clozapine is safe, highly efficacious, but largely underused worldwide (1), possibly due to the need for blood monitoring (3). It is widely encouraged to use it in an earlier phase of psychosis and possibly for more patients (32). Studies have suggested its potential efficacy for patients with bipolar disorder and other mental disorders (33). With an enhanced understanding of its pharmacological actions and safety profile in recent years, clozapine indeed has the potential to become one of the most important psychotropic drugs in the world and act as an important instrument for safeguarding population mental health in the foreseeable future. In line with this development, this study is of great importance because it determines the clinical significance of the potential risk of HM by providing reassuring data on a small, barely concerning elevated risk. Given the existing strict requirements for blood monitoring which helps early identify HM development and the rarity of HM, the risk-benefit balance for clozapine pharmacotherapies remains largely unchanged. Subject to further studies to substantiate our findings, special warnings or more restrictive indication for clozapine may not be necessary.*”

(Lines 310-323, Page 9)

“*To conclude, we conducted a territory-wide retrospective cohort study in Hong Kong and identified a rare but evidently elevated risk of HM among clozapine users with schizophrenia compared with olanzapine users. With a small rate difference and existing blood monitoring requirements, no special warnings or tightening of indication are strictly necessary or recommended. Clinicians should be aware of this consistently observed association but noting its rarity while weighing it against the benefits of clozapine pharmacotherapies.*”

(Lines 359-364, Page 10)

-----------------------------------------------------------

**Reviewer #4:**

**4.1.	“This important study substantiates findings from earlier Finnish and US case-control studies. The study provides incidence estimates that were broadly similar to those from the Finnish cohort study (published alongside the case control study). This and the two previous case-control studies suggest a possible causal association between clozapine and haematological cancer. Although the risk of haematological cancer is rare, this association warrants further attention from clinicians and regulatory bodies. I have minor comments for the authors.”**

**Author Response:**

Thank you very much for your encouraging comment on the importance of this study.

**4.2.	“Abstract: I feel it is somewhat misleading to state in the background that the absolute rate difference is unclear (recommend changing to 'not well established').** **Taipale et al. reported crude incidence rates of 61 cases per 100 000 person-years among clozapine users and 41 cases per 100,000 person-years among users of other antipsychotics. This is alluded to in the introduction (page 3, line 74) and discussion (page 6, line 223). I recommend that authors state these incidence rates from the Finnish study in their introduction.”**

**Author Response:**

Thank you for this comment. We have now replaced “unclear” with “not well-established”. We have also followed your advice to report the incidence rates from the Finnish study in our Introduction as well.

“*Nevertheless, the absolute rate difference is not well-established due to the absence of analytic cohort studies. The clinical significance of such a potential risk remains unclear.*”

(Lines 42-43, Page 2)

“*Though there was also a cohort study design in the Finnish study, it was used to estimate the crude incidence rate of HM without multivariable analyses, i.e., 61 versus 41 per 100,000 person-year among clozapine versus other antipsychotic users.*”

(Lines 103-105, Page 4)

**4.3.	“Methods: I recommend the authors provide more data on the daily medication records extracted from the Clinical Management System (page 4, line 104). I understand these data are widely used for pharmacoepidemiological research. However, it is not clear whether the authors analysed prescribing or dispensing data. I assume that many patients were community-dwelling and prescribed clozapine in hospital outpatient clinics. How was daily medication use ascertained for these patients?”**

**Author Response:**

Thank you for this comment, allowing us an opportunity to clarify this matter. Prescribing records were used in this database but nearly 100% of the drugs prescribed by clinicians in the public healthcare sector in Hong Kong are dispensed in the same facilities. For community dwelling patients, it is indeed challenging to ascertain their daily use. Like other real-world pharmacoepidemiologic studies, our current approach is to assume perfect adherence. However, the observed association is unlikely influenced by non-adherence because we estimated a positive association between clozapine and hematological malignancy, not negative, and the prevalence of non-adherence should arguably be higher in clozapine users because of the widespread perception of clozapine as a hazardous drug according to previous research. We have now added this point as one of the potential limitations in the revised text.

“*Similar with other observational electronic health record studies, we assumed the absence of a disease or medication if no relevant record was identified for the patients in the database.*”

(Lines 201-202, Page 6)

“*Third, there are unobserved covariates such as socioeconomic status and lifestyle factors that were not included in this study. Notably, adherence data is not available despite the high accuracy of the prescription records.*”

(Lines 338-341, Page 9)

**4.4.	“Methods: If I understand correctly, a patient needed to use clozapine or olanzapine for at least 90 days to be considered a user (page 4, line 120). Were patients prescribed/dispensed clozapine or olanzapine for periods less than 90 days excluded from the analyses? If so, how many patients were excluded?”**

**Author Response:**

Thank you for this comment on the duration of clozapine and olanzapine use. Yes, indeed we excluded patients who used clozapine or olanzapine for less than 90 days to avoid including short-term users. In our database, there were 236 patients who used clozapine for less than 90 days but would otherwise be eligible for inclusion in the cohort. For olanzapine the number was 840.

**4.5.	“Results: I recommend the authors report the median clozapine and olanzapine dose over the duration of the follow-up. The Finnish study reported that the association with haematological cancer was dose-dependent (based on cumulative defined daily doses). This may be important for interpreting the association in the present study because clozapine prevalence and dose may differ between countries (e.g. see Bachmann et al. Acta Psychiatr Scand 2017). The authors acknowledge that 'dosage of treatment was not standardized during the course of treatment…' (page 8, line 293). However, it would still be interesting to know the median prescribed doses in Hong Kong.”**

**Author Response:**

Thank you for this helpful suggestion. We have calculated the prescribed daily dose for clozapine users over the observed duration of use and the median was 581.57 mg/day in our cohort. For olanzapine users, the median was 13.72 mg/day. In a new sensitivity analysis, we removed clozapine users who had a daily dose average lower than the 25^th^ percentile and repeated the main analyses. This analysis was repeated another time excluding clozapine users with a daily dose average lower than the median. We obtained results that reflected a potential dose-response relationship, with the incidence rate ratio estimated at 2.93 (95% CI [2.00, 4.42]) and 3.16 (95% CI [2.17,4.78]) respectively.

“… *vii) we excluded one-fourth, and half of the clozapine users who had the lowest prescribed daily dose average across the duration of use in a repeated set of main analysis to observe any potential dose-response relationship…*”

(Lines 196-198, Page 6)

“*With 25% or 50% of clozapine users with the lowest prescribed daily dose average excluded, we estimated larger IRRs which indicate a potential dose-response relationship, i.e., 2.93 (95% CI [2.00, 4.42]) and 3.16 (95% CI [2.17,4.78]). The median average prescribed daily dose of clozapine was 581.57 mg/day and 13.72 mg/day for olanzapine.*”

(Lines 249-253, Page 7)

“*Subgroup analyses by age and sex, as well as sensitivity analyses, showed highly robust results with a potential dose-response relationship, supporting a comparably strong and highly specific association. Our research hypothesis is therefore well supported by the data.*”

(Lines 262-265, Page 8)

-----------------------------------------------------------

We believe these proposed revisions and additional analyses can sufficiently address the concerns raised by the reviewers. We look forward to hearing from you again.

Respectfully submitted,

Francisco Tsz Tsun Lai, PhD

The University of Hong Kong

[fttlai@hku.hk](mailto:fttlai@hku.hk)

Yi Chai, PhD

Shenzhen University

[ychai@szu.edu.cn](mailto:ychai@szu.edu.cn)
