# Supplementary material for: Rare but elevated incidence of hematological malignancy after clozapine use in schizophrenia: A population cohort study
Source: PLoS Med. 2024 Dec 5;21(12):e1004457. doi: 10.1371/journal.pmed.1004457 (PMC11620352; doi:10.1371/journal.pmed.1004457)
Supplement: Attachment — Submitted filename: Response Letter_final.docx [file pmed.1004457.s005.docx]

October 29^th^, 2024

Dr. Alexandra Tosun

Associate Editor, *PLOS Medicine*

Dear Dr. Tosun,

Thank you very much for your favorable consideration of our revised manuscript. We are grateful for the additional reviewer suggestions and the editorial team’s comments. Please find below our detailed response to each of the specific comments with proposed changes and additional analysis. The revised relevant text has been quoted as appropriate:

-----------------------------------------------------------

**Comment from Academic Editor:**

**A1.	“I agree with the reviewers that the responses are adequate and note that the statistics reviewer has made two further helpful suggestions. The only other comment I would make is that they have overstated the safety of clozapine in their responses - this does not reflect the current view of these medications in relation to other side effects such as weight gain, metabolic syndrome, sedation, hypersalivation. These are not "unsafe" in any sense, but some may lead to increased cardiovascular risks in the longer term.”**

**Author Response:**

Thank you for your positive comment on the revisions to the original manuscript. There are indeed metabolic and other side effects of clozapine which might be indirectly related to an increased risk of cardiovascular events. We have now mentioned them with supporting references in the revised text to avoid overstating the safety profile of clozapine.

*“In the existing literature, there is a consensus that clozapine is highly efficacious, generally safe with known tolerable metabolic and other side effects, e.g., weight gain, gastrointestinal hypomotility, etc. [32, 33], but largely underused worldwide [1], possibly due to the need for blood monitoring [3].”*

(Lines 336-339, Page 9)

-----------------------------------------------------------

**Comments from Reviewers:**

**Reviewer #1**

**R1.1.	“The authors have addressed all issues adequately.”**

**Author Response:**

Thank you very much for your encouraging comment on our revisions to the manuscript.

**Reviewer #2**

**R2.1.	“Thanks for the revised manuscript and responses to my original review. The revised manuscript and replies to my review have clarified most of my original review.”**

**Author Response:**

Thank you very much for your positive overall comment on our revisions to the manuscript.

**R2.2.	“I would suggest for the sub-group analyses (Table 2), the most powerful way to test for heterogeneity is to use an interaction term between olanzapine/clozapine and each of the sub-group variables, rather than examine the estimated IRR per sub-group. The IRR of those aged 65 and older has a confidence interval that overlaps that of the other age-groups, which means there isn't good evidence to establish an absence of effect in this age-group compared to the others. Given the relatively small number (21) of Clozapine users in the 65 age-group this is not surprising, and it would be more robust to say that there wasn't evidence of heterogeneity according to age-group.”**

**Author Response:**

Thank you for these suggestions. We tested the interaction between clozapine use and age groups as well as clozapine use and sex accordingly without identifying any significant differences in the observed risk increase across age groups or between sexes. We followed your suggestion and concluded there was no evidence of heterogeneity of the association between sexes and across age groups.

*“The interaction between demographic subgroups and clozapine use was tested in a combined model to examine any potential effect modification of the association across subgroups.”*

(Lines 203-205, Page 6)

*“However, we identified no significant difference in the risk increase between sexes (p=0.786) or between age groups (p = 0.743 between those aged 45-64 years and those 44 years or younger; p=0.290 between those aged 65 years or older and those 44 years or younger). Hence, there was no evidence of heterogeneity between sexes or across age groups*.”

 (Lines 268-271, Page 7)

**Reviewer #3**

**R3.1.	“The authors have improved the quality of their manuscript substantially and addressed all raised points of the reviewers. This article is important for the field of psychiatry and general medicine because it investigates the risk of hematological malignancies of a certain, last resort drug, namely clozapine. They discuss the weighing of risks thoughtully.”**

**Author Response:**

Thank you very much for recognizing the importance of this study.

**Reviewer #4**

**R4.1.	“Thank you for your comprehensive responses to the reviewers' comments. The authors have provided satisfactorily explanations to all my original comments. However, it would be useful if the authors response to comment 4.3 in the response letter was also included in the manuscript. This is because it may seem unusual to readers not familiar with the Hong Kong health system that nearly all drugs prescribed by clinicians in the public healthcare system are dispensed in public clinics and therefore recorded in clinical management system of the Health Kong Hospital Authority. If I did not know otherwise I might assume that only medications prescribed for hospital inpatients or a small sub-subset of patients who attended public-sector outpatient clinics were included in the analyses.”**

**Author Response:**

Thank you for your positive comments on our response to reviewer comments and this helpful suggestion. We have now provided a brief description of this special characteristic of the Hong Kong public healthcare system in the revised text.

*“Nearly 100% of the drugs prescribed by clinicians in the public healthcare sector in Hong Kong are dispensed in the same facilities, with highly accurate records.”*

(Lines 154-156, Page 5)

-----------------------------------------------------------

**Requests from Editors:**

**E1.	“Please ensure to update the data availability section in the online submission form with the details provided in lines 416-421.”**

**Author Response:**

Thank you for your reminder. We have updated the data availability statement in our online submission.

**E2.	“Please ensure to update the financial disclosure section in the online submission form with the details provided in lines 374-376.”**

**Author Response:**

Thank you. We have updated the financial disclosure statement in our online submission.

**E3.	“1) l.47: When reporting age, please add a unit, such as ‘years’. Please revise throughout the Abstract and main manuscript.”**

**Author Response:**

Thank you. We have now added years as the unit of age in the revised abstract and the main text.

*“We extracted data from a territory-wide public healthcare database from January 2001 to August 2022 in Hong Kong to conduct a retrospective cohort study of anonymized patients aged 18+ years with a diagnosis of schizophrenia who used clozapine or olanzapine (drug comparator with highly similar chemical structure and pharmacological mechanisms) for 90+ days, with at least two prior other antipsychotic use records within both groups.”*

(Lines 49-53, Page 2)

*“Subgroup analyses showed a significantly elevated risk of HM among clozapine users for all sex and age groups, except for those aged 65 years or older despite a comparable weighted IRR (Table 2).”*

(Lines 266-268, Page 2)

**E4.	“2) ll.52-55: The IRR presented in the Abstract is the weighted IRR, while the participant numbers presented here are the unweighted numbers. We feel this may not be clear to readers. Please revise and clarify. A sentence such as, "After applying IPTW, the demographic and clinical characteristics of clozapine users were comparable to those of olanzapine users.”, also might be helpful.”**

**Author Response:**

Thank you for this comment. Wherever applicable, we have now clarified by specifying that the reported IRRs are weighted IRR instead of just IRR to avoid confusion.

*“Clozapine users had a significant weighted IRR of 2.22 (95% confidence interval (CI) [1.52, 3.34]; p<0.001) for HM compared to olanzapine users.”*

(Lines 59-61, Page 2)

*“Results from the Poisson regression models show that compared to olanzapine users, clozapine users had a significantly higher IRR of 2.22 (95% CI [1.52, 3.34], p<0.001) for HM after applying IPTW (Table 2).”*

(Lines 262-264, Page 7)

*“Subgroup analyses showed a significantly elevated risk of HM among clozapine users for all sex and age groups, except for those aged 65 years or older despite a comparable weighted IRR (Table 2).”*

(Lines 266-268, Page 2)

*“The weighted IRR for asthma as a negative control outcome was not significant and close to one (weighted IRR 0.90, 95% CI [0.66,1.21]). Likewise, the weighted IRR for other cancers was estimated at 0.77 (95% CI [0.68, 0.86]). The exclusion of 25% or 50% of clozapine users who had the lowest prescribed daily dose average generated even larger weighted IRRs, which indicate a potential dose-response relationship between clozapine use and HM, i.e., 2.93 (95% CI [2.00, 4.42]) and 3.16 (95% CI [2.17,4.78]).”*

(Lines 274-280, Page 8)

**E5.	“3) l.53: “Both groups were followed up for an average of more than seven years.” – please specify by, for example, providing a median plus interquartile range.”**

**Author Response:**

Thank you. We have now added the median follow-up period together with the 25^th^ – 75^th^ percentile.

*“In total, 9,965 patients with a median follow-up period of 6.99 years (25th – 75th percentile: 4.45-10.32 years) were included, among which 834 were clozapine users.”*

(Lines 56-58, Page 2)

*“Sixth, the follow-up time for the olanzapine group was shorter than that of the clozapine group, but it should likely be long enough to observe and capture the study outcome, with a median follow-up time of 6.99 years (25th-75th percentile: 4.45-10.32 years).”*

(Lines 375-378, Page 10)

**E6. “4) l.54: Please define ‘CI’ at first use.”**

**Author Response:**

Thank you. It is now defined at the first appearance.

*“Clozapine users had a significant weighted IRR of 2.22 (95% confidence interval (CI) [1.52, 3.34]; p<0.001) for HM compared to olanzapine users.”*

(Lines 59-61, Page 2)

**E7.	“5) In the last sentence of the Abstract Methods and Findings section, please describe the main limitation(s) of the study's methodology.”**

**Author Response:**

Thank you. We have inserted the main limitation of our study, i.e., lack of randomization, in the revised abstract.

*“The main limitation of this observational study is the potential residual confounding effects that could have arisen from the lack of randomization in clozapine or olanzapine use.”*

(Lines 64-66, Page 2)

**E8.	“In the final bullet point of 'What Do These Findings Mean?', please include the main limitations of the study in non-technical language.”**

**Author Response:**

Thank you. We have inserted the main limitation of our study, i.e., lack of randomization, in the author summary.

*“As the assignment of clozapine versus olanzapine use in this cohort was not randomized, there may be underlying factors that are related to both clozapine use (versus olanzapine) and HM that gave rise to the observed association but were not considered”*

(Lines 96-98, Page 3)

**E9. “l.124, please change to: “hypothesized””**

**Author Response:**

Thank you. It is now changed to “hypothesized”.

“*We hypothesized an elevated risk of HM following the use of clozapine compared with olanzapine.*”

(Lines 135-136, Page 4)

**E10.	“1) Thank you for providing the RECORD checklist. Please replace the line numbers with paragraph numbers per section (e.g. "Methods, paragraph 1"), since the line numbers of the final published paper may be different from the line numbers in the current manuscript”**

**Author Response:**

Thank you. Paragraph number per section is now used in the RECORD checklist.

**E11.	“2) Figure 1: Please define ‘ICD’ in the figure description and add a unit for age.”**

**Author Response:**

Thank you. ICD is now defined in the figure legend.

*“Figure 1. Flow chart showing cohort selection processes. ICD-9 represents International Classification of Diseases, Ninth Revision.”*

(Lines 585-586, Page 16)

**E12.	“3) Table 1: Please add a unit for age. Please define ‘immunological diseases’ below the table.”**

**Author Response:**

Thank you. We have now added years for age as a unit wherever applicable. All the included immunological diseases have been added to the bottom of Table 1.

**E13.	“4) l.226/Table 1: Please note that the category ‘sex’ should refer to males and females.”**

**Author Response:**

Thank you. All expression of “men” and “women” in manuscript and tables has been replaced with “males” and “females” to refer to the categories of sex.

*“The olanzapine group had a higher proportion of males (54.0%) than females, while clozapine the group had a smaller proportion of males (46.6%).”*

(Lines 239-241, Page 7)

**E14.	“5) Table 2: Please add a unit for age. Please note that the category ‘sex’ should refer to males and females.”**

**Author Response:**

Thank you. We have now added years for age as a unit wherever applicable. All expression of “men” and “women” in manuscript and tables has been replaced with “males” and “females” to refer to the categories of sex.

**E15.**	**“6) Figure 2: We feel that it is not really clear where the numbers at risk are derived from. The numbers are not directly reflected in the top graph which might be difficult for readers to understand, and the numbers are not included in any of the tables (except for the numbers at risk at 0 years). The figure, figure description as well as the methods section provide little detail about the analysis. Please revise and also note that the title should include the word "weighted" and the type of graph. We also suggest discussing the results briefly in the results section.”**

**Author Response:**

Thank you. We have changed the term "number at risk" to "weighted number at risk", as it is from the IPTW. The weighted number at risk represent the patient remaining in the cohort at the corresponding time point who had not experienced the outcome, died, or right-censored due to the end of data availability or clozapine/ olanzapine discontinuation after IPTW.

*“Weighted number at risk represent the number of patients remaining in the cohort who had not experienced HM or right-censored at different time points due to death, drug use discontinuation, or the end of data availability after IPTW.”*

(Lines 592-595, Page 17)

**E16.	“7) ll.252-253: Please add a reference to the relevant table.”**

**Author Response:**

Thank you. We have now referred to Table 2 accordingly.

“*Subgroup analyses showed a significantly elevated risk of HM among clozapine users for all sex and age groups, except for those aged 65 years or older despite a comparable weighted IRR (Table 2)*.”

(Lines 266-268, Page 7)

**E17.	“8) l.255, “Sensitivity analyses yielded similar results (S2 Table).” – we suggest specifying which ones.”**

**Author Response:**

Thank you for your suggestion. We have now stated that, with the exception of the analysis using other cancers and asthma as negative control outcomes, all sensitivity analysis demonstrated consistent outcomes with our main analysis.

*“All sensitivity analyses except those using asthma and other cancers as negative control outcomes yielded similar results with main analysis (S2 Table).”*

(Lines 273-274, Page 8)

**E18.	“9) ll.255-261: When discussing IRR values, please make sure that you indicate whether you are referring to the crude or weighted value since you have provided both in your analysis. Please revise throughout the Abstract and the main manuscript.”**

**Author Response:**

Thank you. We referred to the weighted IRR only and have now specified so accordingly wherever applicable.

*“Results from the Poisson regression models show that compared to olanzapine users, clozapine users had a significantly higher weighted IRR of 2.22 (95% CI [1.52, 3.34], p<0.001) for HM after applying IPTW (Table 2).”*

(Lines 262-264, Page 7)

*“Subgroup analyses showed a significantly elevated risk of HM among clozapine users for all sex and age groups, except for those aged 65 years or older despite a comparable weighted IRR (Table 2).”*

(Lines 266-268, Page 7)

*“The weighted IRR for asthma as a negative control outcome was not significant and close to one (weighted IRR 0.90, 95% CI [0.66,1.21]). Likewise, the weighted IRR for other cancers was estimated at 0.77 (95% CI [0.68, 0.86]). The exclusion of 25% or 50% of clozapine users who had the lowest prescribed daily dose average generated even larger weighted IRRs, which indicate a potential dose-response relationship between clozapine use and HM, i.e., 2.93 (95% CI [2.00, 4.42]) and 3.16 (95% CI [2.17,4.78]).”*

(Lines 274-280, Page 8)

**E19.	“10) S2 Table: Please note that in several columns you have indicated that a p-value is included when none is provided.”**

**Author Response:**

Thank you. We have removed “*P*-value” in the column headings in S2 Table.

**E20.	“1) Please remove any subheadings from the Discussion section including the ‘Conclusion’ subheading.”**

**Author Response:**

Thank you. We have removed all the sub-headings in the Discussion section.

**E21.	“2) ll.369-371, please change to: “With a small rate difference and existing blood monitoring requirements, special warnings or tightening of indication may not be strictly necessary or recommended.””**

**Author Response:**

Thank you for the suggestion. We have made this change accordingly.

*“With a small rate difference and existing blood monitoring requirements, special warnings or tightening of indication may not be strictly necessary or recommended.”*

(Lines 390-392, Page 10)

**E22.	“Please ensure that journal name abbreviations match those found in the National Center for Biotechnology Information (NCBI) databases (**[**http://www.ncbi.nlm.nih.gov/nlmcatalog/journals**](http://www.ncbi.nlm.nih.gov/nlmcatalog/journals)**), and are appropriately formatted and capitalised. For example, in reference [21], ‘The Lancet HIV’ should be ‘Lancet HIV’ or in reference [24], ‘International Journal of Psychiatry in Clinical Practice’ should be ‘Int J Psychiatry Clin Pract’.”**

**Author Response:**

Thank you. References are now properly formatted.

**E23.	“****In the published article, supporting information files are accessed only through a hyperlink attached to the captions. For this reason, you must list captions at the end of your manuscript file. You may include a caption within the supporting information file itself, as long as that caption is also provided in the manuscript file. Do not submit a separate caption file. When SI files are contained with a single file: Please label the file as ‘S1 Supporting Information’. Please apply alphabetical labelling to each table and figure contained within the S1 file. For example, ‘Fig A’ to ‘Fig Z’ and ‘Table A’ to ‘Table Z’. Plain text does not need to be labelled and can just be given a title as necessary. For example, ‘Statistical Analysis Plan’. Please cite tables/figures as ‘Fig A in S1 Supporting Information’ and/or ‘Table A in S1 Supporting Information’, for example. Please cite plain text as, ‘Statistical Analysis Plan in S1 Supporting Information’, for example. When SI files are uploaded as separate files: Please label tables as ‘S1 Table’ (so on) and figures as ‘S1 Fig’ (and so on). Any additional documents (protocols/analysis plans etc.) can be labelled as ‘S1 Protocol’, for example. Please cite items as exactly as labelled.”**

**Author Response:**

Thank you for your reminder. We have included the list of captions at the end of the manuscript and uploaded the SI files separately.

**E24.	“To help us extend the reach of your research, please provide any X (formerly known as Twitter) handle(s) that would be appropriate to tag, including your own, your co-authors’, your institution, funder, or lab. Please enter in the submission form any handles you wish to be included when we post about this paper.”**

**Author Response:**

Thank you. We have provided our X (Twitter) handles in our online submission accordingly.

-------------------------------------------------------------------------------------

We believe these proposed revisions and additional analyses can sufficiently address the concerns raised by the reviewers and satisfy the editorial requests. We look forward to hearing from you again.

Respectfully submitted,

Francisco Tsz Tsun Lai, PhD

The University of Hong Kong

[fttlai@hku.hk](mailto:fttlai@hku.hk)

Yi Chai, PhD

Shenzhen University

[ychai@szu.edu.cn](mailto:ychai@szu.edu.cn)
