# Supplementary material for: Rare but elevated incidence of hematological malignancy after clozapine use in schizophrenia: A population cohort study
Source: PLoS Med. 2024 Dec 5;21(12):e1004457. doi: 10.1371/journal.pmed.1004457 (PMC11620352; doi:10.1371/journal.pmed.1004457)
Supplement: Attachment — Submitted filename: Response Letter_final.docx [file pmed.1004457.s006.docx]

November 5^th^, 2024

Dr. Alexandra Tosun

Associate Editor, *PLOS Medicine*

Dear Dr. Tosun,

Thank you very much for your further feedback on the revised manuscript. Please find below our detailed response. The revised relevant text has been quoted as appropriate:

-----------------------------------------------------------

**Comment from Academic Editor:**

**A1.	“The response to the point about the adverse effects of clozapine is brief and rather superficial. They now state: “In the existing literature, there is a consensus that clozapine is highly efficacious, generally safe with known tolerable metabolic and other side effects, e.g., weight gain, gastrointestinal hypomotility, etc. [32, 33], but largely underused worldwide [1], possibly due to the need for blood monitoring [3].” (Lines 336-339, Page 9) The authors should revise this to reflect current evidence on cardiometabolic adverse effects, which are associated with type 2 diabetes, and sedation (which is not mentioned), which is also a major concern for patients.”**

**Author Response:**

Thank you for your further comment on the overstatement of the safety profile of clozapine. We have now cited a much wider variety of safety concerns that may have contributed to the limited use of clozapine in the revised text. We have also toned down the safety of clozapine throughout the text as appropriate.

*“Clozapine is widely regarded as a highly efficacious psychotropic drug that is largely underused worldwide.”*

(Lines 43-44, Page 2)

*“Clozapine is a second-generation antipsychotic agent widely regarded as a highly efficacious psychotropic drug, with the lowest all-cause mortality rate observed compared with other antipsychotics [1, 2].”*

(Lines 101-103, Page 4)

*“In the existing literature, there is a consensus that clozapine is highly efficacious, but largely underused worldwide [1]. Potentially life-threatening adverse effects, such as cardiovascular events, type 2 diabetes, seizure, as well as blood dyscrasias and agranulocytosis/ granulocytopenia, in addition to bothersome side effects, such as weight gain, sedation, nausea and hypersalivation [32, 33], may have contributed to the limited use of it. Nevertheless, with appropriate risk mitigation strategies in place, clozapine is widely encouraged for earlier phases of psychosis and possibly for more patients [34].”*

(Lines 336-342, Page 9)

-----------------------------------------------------------

**Comments from Editors:**

**E1.	“We feel that the Academic Editor's comment has not been satisfactorily addressed as you have understated the adverse effects of clozapine by describing the side effect as "tolerable". For example, in reference [32], the authors discuss the "potentially life-threatening adverse effects which deserve special attention", which we believe is a more accurate statement and what the Academic Editor was pointing to.”**

**Author Response:**

Thank you for this further comment on the overstated safety profile of clozapine. We have now removed “tolerable” and cited a much wider variety of safety concerns that may have contributed to the limited use of clozapine in the revised text. We have also toned down the safety of clozapine throughout the text as appropriate.

*“Clozapine is widely regarded as a highly efficacious psychotropic drug that is largely underused worldwide.”*

(Lines 43-44, Page 2)

*“Clozapine is a second-generation antipsychotic agent widely regarded as a highly efficacious psychotropic drug, with the lowest all-cause mortality rate observed compared with other antipsychotics [1, 2].”*

(Lines 101-103, Page 4)

*“In the existing literature, there is a consensus that clozapine is highly efficacious, but largely underused worldwide [1]. Potentially life-threatening adverse effects, such as cardiovascular events, type 2 diabetes, seizure, as well as blood dyscrasias and agranulocytosis/ granulocytopenia, in addition to bothersome side effects, such as weight gain, sedation, nausea and hypersalivation [32, 33], may have contributed to the limited use of it. Nevertheless, with appropriate risk mitigation strategies in place, clozapine is widely encouraged for earlier phases of psychosis and possibly for more patients [34].”*

(Lines 336-342, Page 9)

-------------------------------------------------------------------------------------

We believe these proposed revisions can sufficiently address the concerns raised by the academic editor. We look forward to hearing from you again.

Respectfully submitted,

Francisco Tsz Tsun Lai, PhD

The University of Hong Kong

[fttlai@hku.hk](mailto:fttlai@hku.hk)

Yi Chai, PhD

Shenzhen University

[ychai@szu.edu.cn](mailto:ychai@szu.edu.cn)
